# Dosimetric impact of adding non‐coplanar arcs for scalp‐avoidance whole‐brain irradiation with volumetric‐modulated arc radiotherapy on scalp dose reduction in pediatric patients with medulloblastomas

**DOI:** 10.1002/acm2.14189

**Published:** 2023-11-02

**Authors:** Daichi Torizuka, Megumi Uto, Takashi Mizowaki

**Affiliations:** ^1^ Department of Radiation Oncology and Image‐applied therapy Kyoto University Graduate School of Medicine Kyoto Japan

**Keywords:** medulloblastoma, non‐coplanar arc, permanent alopecia, scalp‐avoidance whole‐brain irradiation, volumetric‐modulated arc therapy

## Abstract

**Purpose:**

We performed scalp‐avoidance whole‐brain irradiation with volumetric‐modulated arc therapy (SAWB‐VMAT) as a component of craniospinal irradiation. In SAWB‐VMAT with two coplanar arcs, radiation oncologists and medical physicists sometimes experience difficulty in reducing the dose to the scalp to below the cut‐off equivalent dose in 2 Gy per fraction (assuming α/β = 2) to 50% (EQD50%_scalp_). To investigate the advantage of adding coplanar or non‐coplanar arcs in reducing the dose to the scalp in SAWB‐VMAT, we conducted a planning study to compare the EQD50%_scalp_, the dose to other organs at risk (OARs), and target coverage in VMAT with two coplanar arcs (Co2arcVMAT), VMAT with three coplanar arcs (Co3arcVMAT), and VMAT with two coplanar and two non‐coplanar arcs (NcVMAT).

**Methods:**

Co2arcVMAT, Co3arcVMAT, and NcVMAT plans were created for 10 pediatric patients with medulloblastoma. The planned target volume (PTV) included the regions of the whole brain, cervical spinal cord, cerebrospinal fluid space, and intervertebral foramen. The EQD50%_scalp_ was evaluated separately for four areas (top, back, left, and right) in each case. The prescribed dose for the PTV was 35.2 Gy in 22 fractions.

**Results:**

The median EQD50%_scalp_ of the top area was 21.9 , 22.1 , and 18.3 Gy for Co2arcVMAT, Co3arcVMAT, and NcVMAT, respectively. The EQD50%_scalp_ of the top area was significantly reduced in NcVMAT compared to those in Co2arcVMAT and Co3arcVMAT (*p* < 0.05). The median EQD50%_scalp_ of the top area for NcVMAT was < 19.9 Gy, which is the cut‐off dose for severe permanent alopecia. There were no significant differences in EQD50%_scalp_ in the three other areas, the dose to other OARs, or the dose coverage of PTV among the three techniques.

**Conclusion:**

NcVMAT could reduce the EQD50%_scalp_ of the top area below the cut‐off dose of 19.9 Gy. NcVMAT appears to be a promising treatment technique for SAWB‐VMAT.

## 1 INTRODUCTION

The overall survival of patients with medulloblastoma has dramatically improved owing to advances in platinum‐based high‐dose chemotherapy, surgery, and radiotherapy. More than half of the patients with medulloblastoma can be long‐term survivors and return to the social community.^1^, ^2^, ^3^ Advances in multidisciplinary treatment have improved the prognosis of medulloblastoma, and permanent alopecia has been highlighted as a significant late adverse effect caused by dose‐intensive chemotherapy and cranial irradiation, especially in pediatric patients with medulloblastoma.^4^, ^5^

Permanent alopecia can lead to negative self‐esteem and prevent active participation in social activities. It also results in impaired health‐related quality of life (QoL), and celibacy.^6^ Therefore, we performed scalp‐avoidance whole‐brain irradiation with volumetric‐modulated arc therapy (SAWB‐VMAT) as a component of craniospinal irradiation (CSI) to prevent permanent alopecia. It has been previously reported that SAWB‐VMAT significantly reduced the scalp equivalent doses in 2 Gy per fraction (assuming α/β = 2) to 50% of the areas (EQD50%_scalp_) compared to conventional whole‐brain radiotherapy (WBRT), and the estimated cut‐off EQD50%_scalp_ to reduce the incidence of severe permanent alopecia was 19.9 Gy.^7^

SAWB‐VMAT is typically delivered using two coplanar arcs. Despite using SAWB‐VMAT, radiation oncologists and medical physicists sometimes experience difficulty in reducing the dose to the scalp to below 19.9 Gy, which is the estimated cut‐off EQD50%_scalp_ that could reduce the incidence of severe permanent alopecia. Adding non‐coplanar arcs appears to be one potential solution to improve dose distribution and further reduce the dose to the scalp while maintaining target coverage and conformity. The utility of using non‐coplanar arcs in VMAT plans has already been reported for head and neck cancers, and intracranial tumors, including nasopharyngeal cancer, craniopharyngiomas, and frontal to temporal high‐grade glioma.^8^, ^9^, ^10^ Adding non‐coplanar arcs is useful for local irradiation of the primary tumor as well as for hippocampal avoidance in WBRT. Sprowls et al. showed that adding non‐coplanar arcs via HyperArc (Varian Medical Systems, Palo Alto, California, USA) could reduce the dose to the hippocampus in WBRT planning.^11^ However, no study has investigated the utility of adding non‐coplanar arcs to reduce the dose to the scalp and prevent permanent alopecia in SAWB‐VMAT. On the other hand, increasing the number of coplanar arcs is known to improve dose distribution in VMAT planning.^12^

Although it appears that VMAT with three coplanar arcs may also improve dose distribution and reduce the dose to the scalp compared to that with only two coplanar arcs in SAWB‐VMAT, there is no report comparing the dose distribution of the two methods.

Therefore, to investigate the advantage of adding non‐coplanar arcs or other coplanar arc to SAWB‐VMAT, we conducted a planning study to compare the target coverage, EQD50%_scalp_, and the dose to other organs at risk (OARs) in VMAT with two coplanar arcs (Co2arcVMAT), VMAT with three coplanar arcs (Co3arcVMAT), and VMAT with two coplanar and two non‐coplanar arcs (NcVMAT).

## 2 METHODS

The study was conducted in accordance with the principles of the Declaration of Helsinki. This study was approved by the institutional ethical review board of our hospital (approval number: R1048‐1). Written informed consent was obtained from all patients enrolled in this study.

### 2.1 Study population

Ten pediatric patients (median age: 8.9 years, range: 4.7−12.9 years) were included in the study. All patients were diagnosed with medulloblastoma and underwent CSI at our institution between June 2011 and August 2021.

### 2.2 Target and OARs delineation

The patients were immobilized in the supine position with thermoplastic masks during the computed tomography (CT) simulation. Contouring and treatment planning were performed using Eclipse version 16.1 (Varian Medical Systems, Palo Alto, California, USA). The CT images had a slice thickness of 2.0 mm and were acquired using a Light Speed RT scanner (GE Healthcare, Milwaukee, Wisconsin, USA). For Co2arcVMAT, Co3arcVMAT, and NcVMAT planning, we defined the contours of the target volume as follows: the clinical target volume (CTV) included regions of the whole brain, cervical spinal cord, cerebrospinal fluid space, and intervertebral foramen. In addition, the CTV included a 5 mm margin on the bilateral optic nerves to compensate for movement. To account for patient movement and setup errors, a 3 mm margin was added to the cranial region of the CTV to create the planned target volume (PTV). Because the setup error was larger in the cervical region than in the cranial region, a 5 mm margin was added to the cervical region of the CTV to obtain the PTV.

In boost planning, the area of the whole posterior fossa was defined as CTVboost, and a 3 mm margin was added to CTVboost to create the PTVboost.

Hair follicles have an approximate depth of 4.5 mm from the body surface, and the scalp was defined as the inner 5 mm region of the body contour as an OAR for estimating the risk of permanent alopecia, as suggested by Roberge et al. and Mahadevan et al.^4^, ^13^, ^14^

The body thickness of the top cranial region is thinner than the other cranial regions and is likely prone to permanent alopecia due to the higher doses received from conventional whole‐brain irradiation.^7^ In addition, we considered it necessary to evaluate the scalp dose to the back region separately to consider the impact of boost irradiation for the tumor bed or the posterior fossa on the scalp dose to the back region.

Since evaluating the dose for the entire scalp does not allow for the evaluation of the scalp doses in different areas with different body thicknesses, we further divided the scalp into four areas (top, back, left, and right) according to the method described in a previous report on SAWB‐VMAT.^7^

To ensure objectivity and reproducibility, the four areas of the scalp were defined according to anatomical structures such as the earlobe and supraorbital border. In detail, the top area was defined as the scalp area above the supraorbital border. In the region caudal to the top area, the bilateral frontal region of the ear lobe was defined as the right and left temporal area. Finally, the posterior area of the ear lobe was defined as the back area.

The contours of the eye, lens, hypopharynx, cochlea, thyroid, oral cavity, and parotid glands were also drawn.

### 2.3 Treatment planning of SAWB‐VMAT and boost plan

Co2arcVMAT, Co3arcVMAT, and NcVMAT plans were created for each of the 10 patients. A 6‐megavolt beam, delivered by TrueBeam (Varian Medical Systems) was used in all patients. The dose was calculated using the Acuros XB dose calculation algorithm (ver. 16.1). The prescribed dose for the PTV was 35.2 Gy in 22 fractions, and each plan was normalized to V_90_ = 99% (i.e., 99% of the PTV was covered by 90% of the prescribed dose).

In the boost plan, the prescribed dose for the PTVboost was 19.8 Gy in 11 fractions, and each plan was normalized to D_50%_ (i.e., 50% of the PTVboost was covered by the prescribed dose).

### 2.4 Beam arrangement and optimization: Co2arcVMAT and Co3arcVMAT planning

Co2arcVMAT plans consisted of double coplanar arcs. One arc was rotated clockwise from 181° to 179° with a collimator angle of 350°. The other arc was rotated counter‐clockwise from 179° to 181°, and the collimator angle was 10°. On the other hand, Co3arcVMAT plans were created by adding one coplanar arc that rotated clockwise from 181° to 179° with the collimator angle of 350° to the Co2arcVMAT plans.

In Co2arcVMAT and Co3arcVMAT plans, optimization was performed until the results met the following criteria: the D_2%_ (D_X%_ is the dose to X% of the target or OAR volume.) of the PTV was set to < 110% of the prescribed dose, and the doses to the scalp and other OARs were reduced as much as possible while maintaining the dose coverage of the PTV. For the scalp, the EQD50%_scalp_ for each area was equally and maximally reduced to be below 19.9 Gy, which is the cut‐off dose to reduce the incidence of severe permanent alopecia. To further reduce the dose to the top area, there was no attempt to loosen the dose constraints in other scalp areas.

### 2.5 Beam arrangement and optimization: NcVMAT planning

NcVMAT plans were created using two coplanar and two non‐coplanar arcs.

**FIGURE 1:**
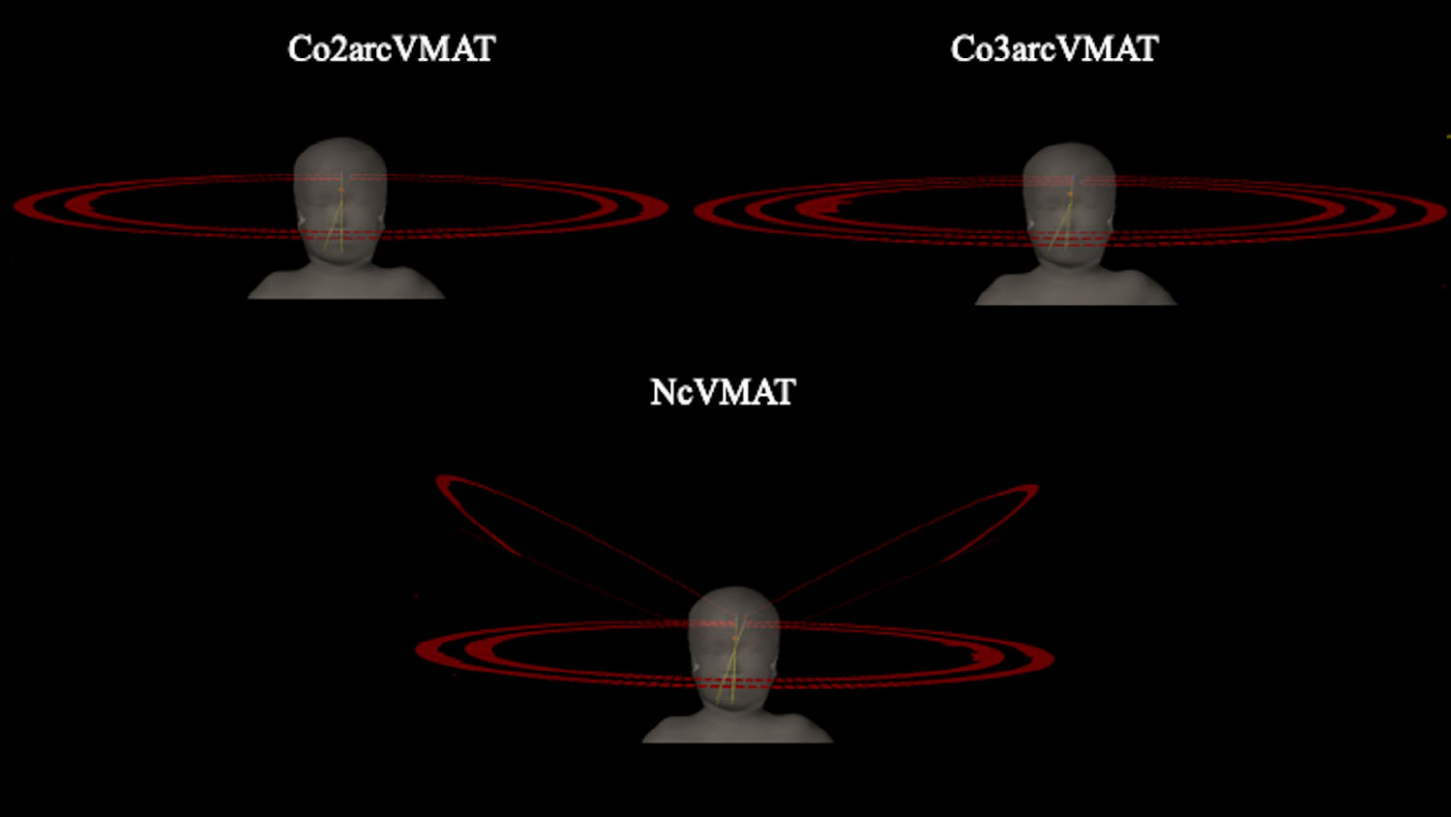
Arc arrangement of volumetric‐modulated arc therapy (VMAT) with two coplanar arcs (Co2arcVMAT), VMAT with three coplanar arcs (Co3arcVMAT), and VMAT with two coplanar and two non‐coplanar arcs (NcVMAT).

The beam arrangement of the coplanar arcs in the NcVMAT plans was identical to that in the Co2arcVMAT plans. Non‐coplanar arcs were placed at couch angles of 330° and 30° to avoid the shoulders (Figure 1). One arc rotated clockwise from 181° to 320°, and the other arc rotated counterclockwise from 179° to 40°, in which both eyes were not directly irradiated. The collimator angles were set to 350° and 10°, respectively.

Optimization was performed to meet the same criteria as those used in the Co2arcVMAT and Co3arcVMAT plans (see the previous section).

### 2.6 Beam arrangement and optimization: Boost planning

Boost plans were created using one coplanar and two non‐coplanar arcs.

The coplanar arc was rotated clockwise from 181° to 179°, the collimator angle was set at 350°, and the avoidance sector was set from 310° to 50° to avoid direct irradiation to the eye. The beam arrangement of the non‐coplanar arcs in the boost plans was identical to that in the NcVMAT plans.

Optimization was performed to meet the same criteria as those used in the Co2arcVMAT, Co3arcVMAT, and NcVMAT plans (see the previous section).

### 2.7 Plan evaluation

To evaluate the target coverage, we assessed the D_2%_, D_50%_, D_98%_, and V_95%_ of the PTV. V_95%_ refers to the volume of the PTV irradiated with 95% of the prescribed dose. In addition, target conformity was evaluated using the Ian Paddick Conformity Index (IP‐CI), which was defined as VPTV(90)^2^/[V(90) × VPTV], where VPTV(90) is the volume of the PTV receiving 90% of the prescribed dose.^15^

Target homogeneity was evaluated using the International Commission on Radiation Units and Measurements homogeneity index (HI).^16^ The HI is defined as (D_2%_‐ D_98%_)/ D_50%_, where D_2%_, D_98%_, and D_50%_ are the doses covering 2%, 98%, and 50% of the PTV, respectively.

With regard to the dose to the scalp, we assessed the equivalent dose in 2 Gy per fraction (assuming α/β = 2) to 50% of the scalp (EQD50%_scalp_) in four areas for each patient. For the other OARs, we estimated D_2%_ and D_50%_ of the hypopharynx, thyroid, and oral cavity. In addition, D_50%_ of the cochlea and parotid were estimated, and D_2%_ of the eyes and lenses were evaluated.

The monitor unit (MU) and beam‐on time were calculated.

### 2.8 Statistical analysis

All statistical analyses were performed using EZR software (Saitama Medical Center, Jichi Medical University; http://www.jichi.ac.jp/saitamasct/SaitamaHP.files/manual.html), which is a graphical user interface for R (R Foundation for Statistical Computing, Vienna, Austria, version 3.6.3). EZR is a modified version of R commander version 2.6‐2 and is used for biostatistical evaluations.^17^ The Co2arcVMAT, Co3arcVMAT, and NcVMAT plans were compared across the entire cohort using the Kruskal–Wallis test with the Steel–Dwass post hoc test, as appropriate. Statistical significance was set at *p* < 0.05.

## 3 RESULTS

### 3.1 The comparison of EQD50%_scalp_

Table 1 summarizes the EQD50%_scalp_ separated into four areas. The median EQD50%_scalp_ of the top area was 21.9, 22.1, and 18.3 Gy in Co2arcVMAT, Co3arcVMAT, and NcVMAT, respectively. The EQD50%_scalp_ of the top area in NcVMAT was significantly reduced than those of the other two modalities (*p* < 0.05).

**TABLE 1:** EQD50%_scalp_ separated into four areas.

Area/EQD50%_scalp_ (Gy)	Co2arcVMAT	Co3arcVMAT Median (IQR)	NcVMAT	*p*
Rt temporal	15.9 (12.0−18.7)	15.6 (11.7−18.2)	15.2 (11.9−16.6)	0.88
Lt temporal	16.3 (11.7−17.7)	16.2 (11.9−17.3)	16.0 (11.7−16.7)	0.85
Top	21.9 (19.2−22.9)	22.1 (19.2−23.0)	18.3 (16.4−20.0)	< 0.05
Back	20.1 (19.2−21.8)	20.6 (19.2−21.4)	19.2 (17.9‐20.5)	0.55

In the top area, *p* value was 0.99, < 0.05, < 0.05 in Co2arcVMAT vs. Co3arcVMAT, Co2arcVMAT vs. NcVMAT, and Co3arcVMAT vs. NcVMAT, respectively.

Abbreviations: Co2arcVMAT, volumetric‐modulated arc therapy using two coplanar arcs; Co3arcVMAT, volumetric‐modulated arc therapy using three coplanar arcs; EQD50%_scalp,_ the scalp equivalent doses in 2 Gy per fraction (assuming α/β = 2) to 50% of the areas.; IQR, interquartile range; NcVMAT, volumetric‐modulated arc therapy using two coplanar and two non‐coplanar arcs.

**FIGURE 2:**
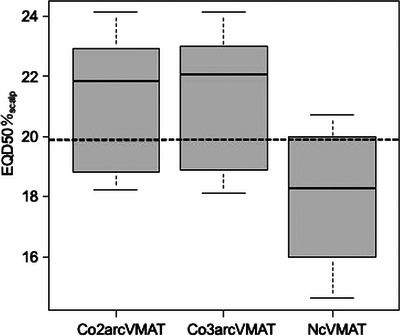
Box plot of the EQD50%_scalp_ of the top area in Co2arcVMAT, Co3arcVMAT, and NcVMAT. The cut‐off dose of 19.9 Gy is indicated by the dotted line.

In addition, the median EQD50%_scalp_ of the top area for NcVMAT was < 19.9 Gy, which was the cut‐off dose that could reduce the incidence of severe permanent alopecia (Figure 2).

**FIGURE 3:**
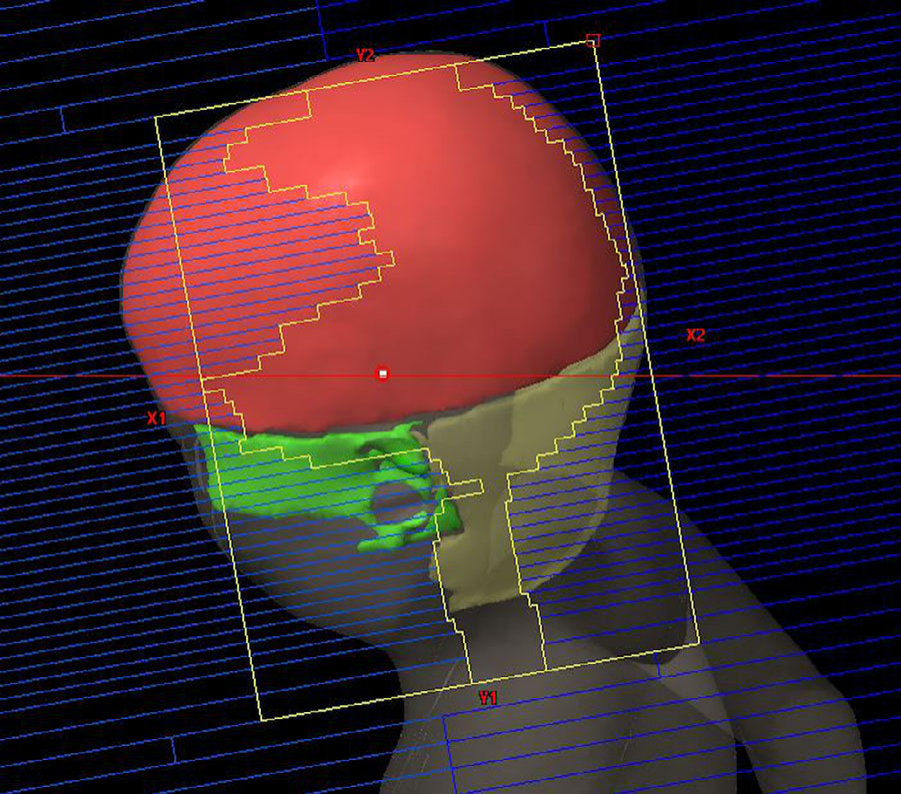
Beams eye view of non‐coplanar beam in NcVMAT.

**FIGURE 4:**
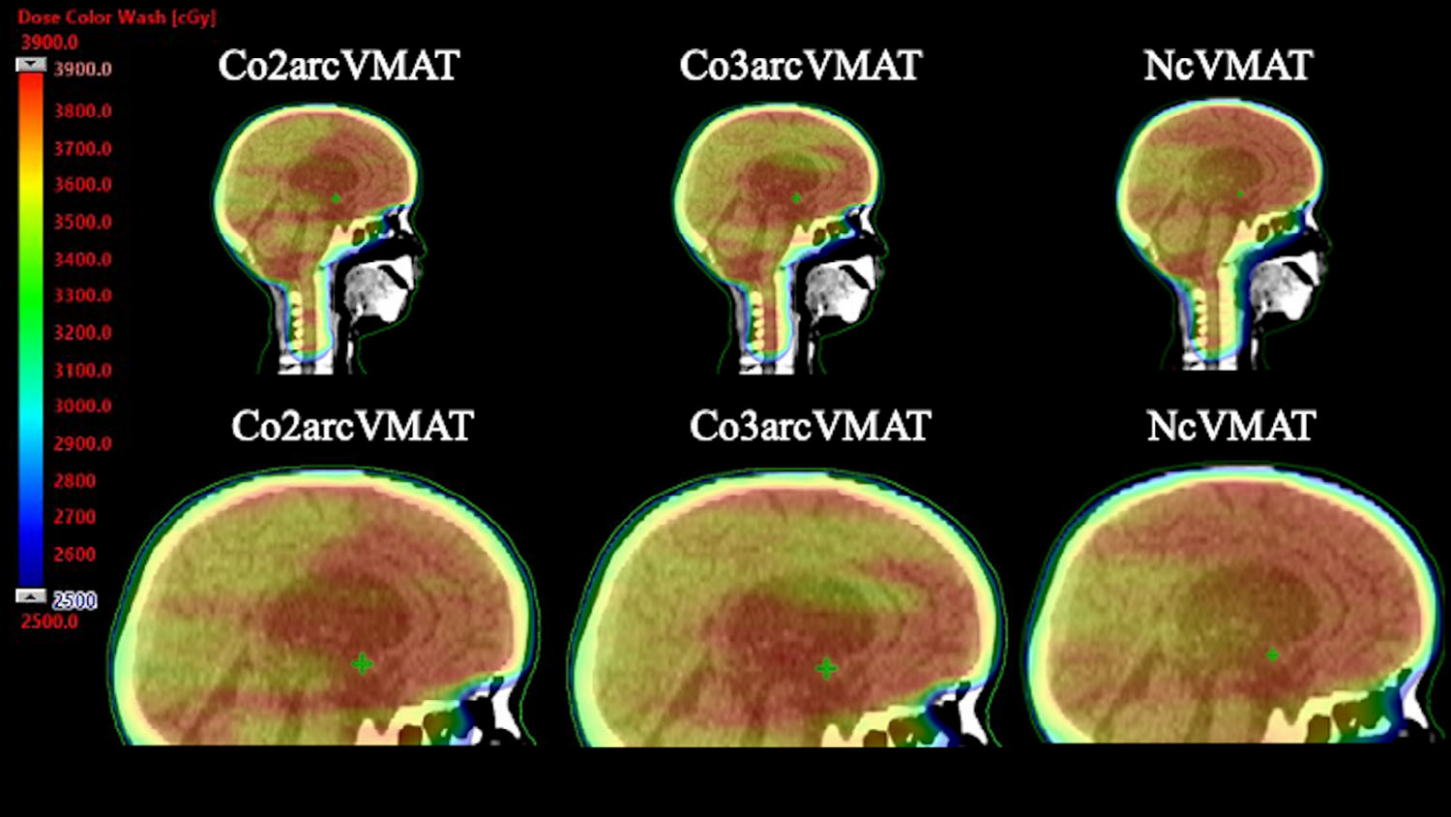
Single sagittal slice showing the dose distribution of each plan in a representative case using Co2arcVMAT, Co3arcVMAT, and NcVMAT. The areas irradiated above 25 Gy are shown.

A representative beam‐eye view of the non‐coplanar beam in NcVMAT is shown in Figure 3. In NcVMAT, the top area of the scalp is shielded from various angles by the multi‐leaf collimator (MLC) and the jaw. A single sagittal slice showing the dose distribution for each modality is shown in Figure 4.

### 3.2 Target coverage and the dose to other OARs

Table 2 summarizes the PTV indices. We found no significant differences among the three techniques in terms of the IP‐CI, ICRU‐HI, and other PTV indices.

**TABLE 2:** Dosimetric indices for the planning target volume.

Index	Co2arcVMAT	Co3arcVMAT Median (IQR)	NcVMAT	*p* Kruskal‐Wallis
IP‐CI	0.64 (0.56−0.66)	0.60 (0.52−0.64)	0.65 (0.62−0.77)	0.26
HI	0.16 (0.15−0.16)	0.15 (0.15−0.16)	0.16 (0.15−0.16)	0.49
D_2%_ (Gy)	38.1 (38.0−38.3)	38.0 (37.8−38.1)	38.0 (37.8−38.2)	0.33
D_98%_ (Gy)	32.3 (32.3−32.4)	32.3 (32.3−32.4)	32.4 (32.3−32.4)	0.73
D_50%_ (Gy)	35.6 (35.4−35.6)	35.5 (35.3−35.6)	35.6 (35.5−36.1)	0.52
V_95_ (%)	94.8 (94.3−95.2)	94.5 (94.2−94.8)	95.0 (94.6−95.4)	0.36

Abbreviations: Co2arcVMAT, volumetric‐modulated arc therapy using two coplanar arcs; Co3arcVMAT, volumetric‐modulated arc therapy using three coplanar arcs; D_2%_, dose to 2% of the volume; D_50%_, dose to 50% of the volume, V_95_, percentage of the volume which was covered by 95% of the prescribed dose.; D_98%_, dose to 98% of the volume; HI, homogeneity index; IP‐CI, Ian Paddick conformity index; IQR, interquartile range; NcVMAT, volumetric‐modulated arc therapy using two coplanar and two non‐coplanar arcs.

No significant differences regarding the dose to other OARs, excluding the scalp, were observed among the three modalities (Table 3).

**TABLE 3:** Summary of the OAR doses (excluding scalp).

Structure/index (Gy)	Co2arcVMAT	Co3arcVMAT Median (IQR)	NcVMAT	*p*
Rt eye				
D_2%_	27.5 (26.6−28.3)	28.0 (26.1−28.7)	28.6 (27.1−30.6)	0.50
Lt eye				
D_2%_	26.4 (25.6−27.7)	27.2 (26.5−27.8)	28.1 (27.0−31.4)	0.15
Rt lens				
D_2%_	10.3 (9.7−11.5)	10.7 (10.0−12.1)	10.9 (9.5−11.8)	0.75
Lt lens				
D_2%_	10.0 (9.4−11.3)	10.2 (9.6−11.4)	10.6 (9.7−11.2)	0.87
Rt cochlea				
D_50%_	40.0 (39.5−40.1)	39.9 (39.6−40.1)	40.3 (39.5−40.7)	0.52
Lt cochlea				
D_50%_	41.8 (41.5−42.6)	41.9 (40.8−42.5)	42.3 (41.8−42.6)	0.65
Rt Parotid				
D_50%_	13.6 (12.4−14.4)	13.5 (12.3−14.8)	14.0 (12.5−14.8)	0.83
Lt Parotid				
D_50%_	12.7 (11.8−14.5)	12.7 (11.6−14.4)	13.2 (11.8−14.5)	0.90
Hypopharynx				
D_2%_	19.2 (16.9−19.8)	18.6 (17.3−21.3)	19.2 (17.4−21.6)	0.77
D_50%_	10.5 (10.3−10.8)	10.6 (10.5−11.0)	10.7 (10.6−12.8)	0.25
Thyroid				
D_2%_	17.2 (15.9−18.5)	17.9 (16.6−19.1)	17.2 (15.2−18.9)	0.85
D_50%_	11.3 (9.4−11.9)	11.0 (9.3−12.2)	10.7 (9.7−12.4)	0.96
Oral Cavity				
D_2%_	18.6 (16.3−19.5)	19.4 (17.6−20.4)	19.7 (17.7−20.5)	0.51
D_50%_	10.0 (9.0−10.7)	9.9 (9.0−10.8)	10.6 (9.2−11.2)	0.64

Abbreviations: Co2arcVMAT, volumetric‐modulated arc therapy using two coplanar arcs; Co3arcVMAT, volumetric‐modulated arc therapy using three coplanar arcs; D_2%_, dose to 2% of the volume; D_50%_, dose to 50% of the volume.; IQR, interquartile range; NcVMAT, volumetric‐modulated arc therapy using two coplanar and two non‐coplanar arcs.

### 3.3 Monitor units and beam‐on times

The median MU values of Co2arcVMAT, Co3arcVMAT, and NcVMAT were 250.8 [interquartile range (IQR): 246.2 – 259.1)], 260.2 (IQR: 251.6 – 265.1), and 265.4 (IQR: 247.7−282.4), respectively, which were not significantly different (*p* = 0.347). The beam‐on times were 118.0, 177.0, and 164.4 s for Co2arcVMAT, Co3arcVMAT, and NcVMAT, respectively.

## 4 DISCUSSION

We found that the EQD50%_scalp_ of the top area was significantly reduced in NcVMAT compared with those of the other two modalities. The median EQD50%_scalp_ of the top area in NcVMAT was < 19.9 Gy, which is the cut‐off dose that could reduce the incidence of severe permanent alopecia. There were no significant differences among the three techniques in terms of EQD50%_scalp_ in the other three areas, PTV coverage, and the dose to other OARs, except for the scalp.

In the present study, we cannot show statistically significant scalp dose reduction in other areas as well as the top area in NcVMAT with the limited number of cases, and larger studies are needed to determine statistically significant differences.

In the planning of WBRT with VMAT, the advantage of delivering non‐coplanar arcs has been reported in terms of sparing the hippocampus.^11^ However, no studies have investigated the utility of adding non‐coplanar arcs to reduce the dose to the scalp in WBRT with VMAT. Our study showed dose reduction to the scalp of the top area by adding non‐coplanar arcs in NcVMAT. This could be because the trajectory of the nonplanar arcs was tangential to the scalp, and the scalp of the top area was shielded from various angles by the multileaf collimator (MLC) and jaws, as shown in Figure 4.

Using coplanar arcs and adding non‐coplanar arcs enable irradiation from various angles, and NcVMAT appears to provide better dose distribution. To our knowledge, this is the first study to demonstrate the advantage of adding non‐coplanar arcs to WBRT with VMAT to reduce the dose to the scalp.

In our clinical experience, permanent alopecia of the temporal and back areas can be concealed by long hair from the top area and sometimes tends to be unnoticeable. On the other hand, permanent alopecia in the top area may be difficult to conceal, except by wearing a wig. Therefore, we think that preventing permanent alopecia in the top area is especially important to maintain and improve QoL compared with the other three areas.

To consider the junction matching of the SAWB‐VMAT and CSI plan, we did not create plans to take into account the overlap with superior spine fields. This is because the extent and angle of the fan beam in the superior spine field varies from patient to patient. However, based on the dose distribution in the spinal cord irradiation in each patient, it is possible to develop the SAWB‐VMAT plan that modifies the dose gradient in the caudal region. Therefore, junction matching using these VMAT techniques is feasible.

The limitations of this study should be considered. First, the treatment time for NcVMAT was longer than that for Co2arcVMAT and Co3arcVMAT. The beam‐on time was longer for the NcVMAT than for Co2arcVMAT. Moreover, the couch must be rotated to deliver non‐coplanar arcs. As a result, NcVMAT required significantly more time than VMAT with only coplanar arcs. Pediatric patients sometimes require sedation to maintain their treatment position during radiotherapy, and it is important to perform safe and rapid image‐guided radiotherapy when delivering NcVMAT in SAWB‐VMAT. Second, planning NcVMAT may require extra treatment planning time on medical physicists and radiation oncologists. Although non‐coplanar arcs are used in SAWB‐VMAT, it remains relatively difficult to create acceptable SAWB‐VMAT plans that achieve both target coverage and reduce the dose to OARs, including the scalp. To address this issue, the use of artificial intelligence such as RapidPlan could potentially relieve the burden on medical staff. Although we have not quantitatively evaluated treatment times and compared with automated plans, we would like to analyze these issues as a next step.

Finally, this was a dosimetric study involving a small number of patients. It is unclear whether reducing the dose to the scalp using NcVMAT will lead to decreasing the incidence of permanent alopecia.

## 5 CONCLUSION

SAWB‐VMAT with non‐coplanar arcs can reduce the EQD50%_scalp_ of the top area to below the cut‐off dose of 19.9 Gy, which could reduce the incidence of severe permanent alopecia. There were no significant differences among the three techniques regarding EQD50%_scalp_ of the other three areas, target coverage, and the dose to other OARs. This is the first study to demonstrate the advantage of adding non‐coplanar arcs to SAWB‐VMAT in reducing the dose to the scalp in pediatric patients with medulloblastomas.

## AUTHOR CONTRIBUTIONS

Daichi Torizuka performed the study and the statistical analysis and drafted the manuscript. Megumi Uto and Takashi Mizowaki conceived the study, participated in its design and coordination, and also helped to draft the manuscript. All authors read and approved the final manuscript.

## CONFLICT OF INTEREST STATEMENT

No conflicts of interest.

## Data Availability

The data that support the findings of this study are not available.
